# Moderate-Risk Genes for Hereditary Ovarian Cancers Involved in the Homologous Recombination Repair Pathway

**DOI:** 10.3390/ijms231911790

**Published:** 2022-10-04

**Authors:** Akiko Abe, Issei Imoto, Arisa Ueki, Hidetaka Nomura, Hiroyuki Kanao

**Affiliations:** 1 Department of Gynecologic Oncology, Cancer Institute Hospital of JFCR, Tokyo 135-8550, Japan; 2 Aichi Cancer Center Research Institute, Nagoya 464-8681, Japan; 3 Clinical Genetic Oncology, Cancer Institute Hospital, Japanese Foundation for Cancer Research, Tokyo 135-8550, Japan

**Keywords:** epithelial ovarian cancer, germline pathogenic variant, hereditary tumor, homologous recombination repair pathway, moderate risk

## Abstract

Approximately 20% of cases of epithelial ovarian cancer (EOC) are hereditary, sharing many causative genes with breast cancer. The lower frequency of EOC compared to breast cancer makes it challenging to estimate absolute or relative risk and verify the efficacy of risk-reducing surgery in individuals harboring germline pathogenic variants (GPV) in EOC predisposition genes, particularly those with relatively low penetrance. Here, we review the molecular features and hereditary tumor risk associated with several moderate-penetrance genes in EOC that are involved in the homologous recombination repair pathway, i.e., *ATM, BRIP1, NBN, PALB2*, and *RAD51C/D*. Understanding the molecular mechanisms underlying the expression and function of these genes may elucidate trends in the development and progression of hereditary tumors, including EOC. A fundamental understanding of the genes driving EOC can help us accurately estimate the genetic risk of developing EOC and select appropriate prevention and treatment strategies for hereditary EOC. Therefore, we summarize the functions of the candidate predisposition genes for EOC and discuss the clinical management of individuals carrying GPV in these genes.

## 1. Introduction

In 2020, ovarian cancer ranked as the eighth most common cancer in women, with an estimate of almost 320,000 new cases worldwide [1]. More than 70% of cases are diagnosed at advanced stages [2,3], partly because of the delayed onset of disease-specific symptoms and the absence of effective screening tools, which result in high mortality rates despite initial treatment [4]. Approximately 90% of ovarian cancers are epithelial ovarian cancers (EOCs), which can be further classified into five major histological subtypes with different risk factors and molecular characteristics: high-grade serous, low-grade serous, clear cell, endometrioid, and mucinous carcinomas [5,6].

Although the pathogenesis of EOC is not well characterized, several risk factors for developing EOC have been identified, including both acquired environmental and genetic factors. Environmental factors include older age, early menarche or late menopause, smoking, and being overweight or obese [6,7,8]. Moreover, the widespread use of oral contraceptive pills, other reproductive factors such as higher parity (more children), and breastfeeding have been reported as protective factors against EOC [6,8,9]. Chronic inflammation can directly cause DNA damage related to cancer initiation and progression [10,11]. Thus, proinflammatory conditions such as pelvic inflammatory disease [12,13,14] and endometriosis [15,16] are also associated with a risk of developing EOC. Furthermore, up to 20% of EOC cases, particularly high-grade serous carcinomas, may be caused by germline pathogenic variants (GPVs) in various cancer predisposition genes [17,18]. A smaller proportion of other ovarian cancer subtypes are also likely to be related to GPVs in EOC predisposition genes. Some research suggests that oxidative stress during the menstrual cycle may play a role in ovarian tumorigenesis [19]. Moreover, the regulation of hormones, especially estrogen, appears to increase double-strand breaks (DSBs), which may explain tissue specificity [20,21,22].

The known EOC predisposition genes predominantly belong to two different DNA repair pathways [23,24,25,26,27,28,29]. Most EOC predisposition genes, including *BRCA1* and *BRCA2*, are involved in part of the homologous recombination (HR) repair pathway, which governs the error-free DNA repair mechanism. Conversely, the mismatch repair (MMR) genes, such as *MLH1*, *MSH2*, *MSH6*, and *PMS2*, are involved in the MMR pathway, which handles erroneous misincorporations, insertions, and deletions of nucleotides. GPVs in genes encoding proteins important for the HR repair pathway increase the risk of high-grade serous carcinoma, whereas GPVs in MMR genes increase the risk of endometriosis-associated EOC, such as endometrioid and clear cell carcinomas [26,30]. In addition, a number of common variants associated with EOC susceptibility have been identified by genome-wide association studies [31,32]. Thus, the ability to accurately estimate ovarian cancer risk using genetic information from the patient may have crucial implications for EOC management in individuals.

**Figure 1:**
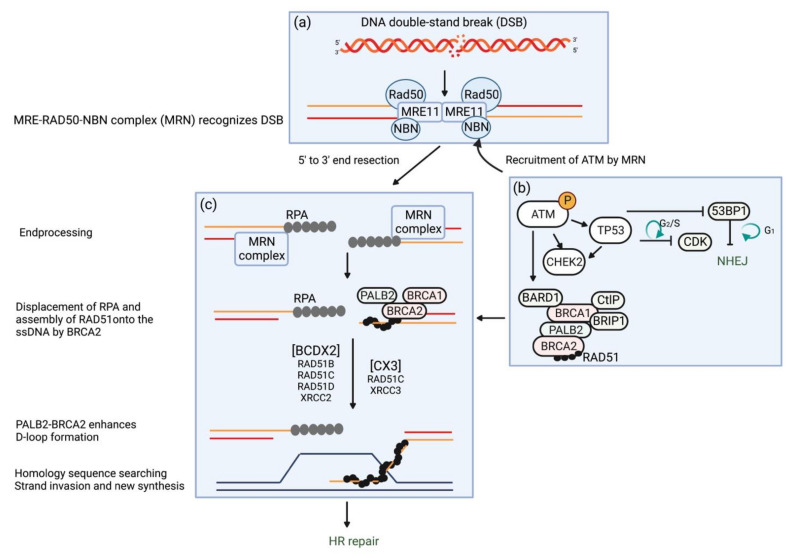
Schematic of DNA double-strand break (DSB) repair by homologous recombination (HR) and key molecules: MRE-RAD50-NBN (MRN) protein complex, ATM, BARD, BRCA1, BRCA2, BRIP1, CHEK2, PALB2, and RAD51C/D. The free DNA ends produced by DSBs are recognized by the MRN protein complex (**a**). The MRN protein complex recruits and activates ATM, which in turn phosphorylates and activates many downstream targets essential for DNA damage repair via nonhomologous end-joining (NHEJ) and HR (**b**,**c**). The replication protein A (RPA) is recruited by the BRCA1–PALB2–BRCA2 effector complex and is loaded on a long 3′ single-stranded DNA (ssDNA) tail to form RAD51–ssDNA nucleofilament. BRCA2 mediates displacement of RPA with RAD51. PALB2-BRCA2 enhances D-loop formation, which is followed by HR repair. The RAD51 paralogs associated in protein complexes (RAD51B-RAD51C-RAD51D-XRCC2 (BCDX2) and RAD51C-XRCC3 (CX3)) participate in the assembly and stabilization of the ssDNA/RAD51 filament and the HR intermediates as well as in the steps downstream of the homology search (not represented) [56].

Several screening tools have been proposed for EOC, such as serial transvaginal ultrasound and serum cancer antigen 125 (CA-125); however, there are no effective secondary prevention strategies for reducing mortality in EOC [3]. Therefore, the only strategy shown to reduce EOC mortality in women with a high risk of developing EOC is primary prevention, such as risk-reducing salpingo-oophorectomy (RRSO) and chemoprevention. For example, in *BRCA1-* and *BRCA2*-associated hereditary breast and ovarian cancer syndromes (HBOC), RRSO decreases the incidence of EOC and reduces mortality [32,33,34,35], whereas the potential benefits and dangers of oral contraceptive pills, a type of chemoprophylaxis for EOC, remain unclear [36]. In contrast to the high-penetrance *BRCA1* or *BRCA2* (*BRCA1/2*) gene, there is still substantial controversy regarding the degree of conferred risks for EOC in individuals with other EOC predisposition genes and whether those risks are sufficiently elevated above the general population to warrant consideration of RRSO [37,38]. Additionally, RRSO has potential negative aspects with regard to women’s health, such as cardiovascular events and osteoporosis. Even though RRSO prevents EOC, there is concern that cardiovascular events caused by RRSO-induced ovarian dysfunction may worsen life expectancy and reduce the quality of life due to menopausal symptoms. For this reason, several clinical trials are currently being conducted to demonstrate the risk-reducing effects of prior risk-reducing salpingectomy (RRS) [38,39,40,41]. Because most EOC-associated genes are involved in one of two DNA repair pathways [42], a fundamental understanding of disease drivers in these pathways will allow us to accurately estimate the genetic risk of developing EOC and select appropriate prevention and treatment strategies for hereditary EOC. Syndromic diseases such as Lynch syndrome, Li–Fraumeni syndrome, Cowden syndrome/PTEN hamartoma tumor syndrome (PHTS), Peutz–Jeghers syndrome, DICER1 syndrome, and rhabdoid tumor predisposition syndrome—which are caused by GPVs in MMR genes, *TP53*, *PTEN*, *STK11*, *DICER1*, and *SMARCB1*/*SMARCA4*, respectively, and may also predispose to EOC [24,43,44]—can be characterized separately as syndromes. In this review, therefore, we mainly focused on candidate EOC predisposition genes involved in the HR repair pathway (Figure 1), summarized their molecular mechanisms of cancer predisposition, and discussed the clinical management of individuals carrying GPVs in each of these genes.

## 2. Predisposition Genes Included in This Study

Previous research has reported many EOC-associated genes. In a recent meta-analysis, Suszynska et al. [45] reported 11 cancer predisposition genes, including *BRCA1* and *BRCA2*, that were significantly associated with the development of EOC. Through a large-scale association analysis, they compared the frequencies of GPVs in a group of approximately 120,000 controls derived from the population-based noncancer Genome Aggregation Database (gnomAD) [45]. These statistically significant EOC-associated genes include *ATM*, *BRIP1*, *BRCA1*, *BRCA2*, *MSH2*, *MSH6*, *NBN*, *PALB2*, *RAD51C*, *RAD51D*, and *TP53*. Liu et al. [30] also identified *ATM*, *BRIP1*, *BRCA1*, *BRCA2*, *MLH1*, *MSH2*, *MSH6*, *PALB2*, *RAD51C*, and *RAD51D* as EOC-associated genes that exhibited a risk beyond that of the general population risk for EOC. By combining these lists with the clinical practice guidelines in oncology of the National Comprehensive Cancer Network (NCCN) [46,47], we selected *ATM*, *BRIP1*, *BRCA1*, *BRCA2*, *NBN*, *PALB2*, *RAD51C*, and *RAD51D* as key EOC-associated genes involved in the HR repair pathway (Figure 1).

Among these genes, we focused on the following six: *ATM*, *BRIP1*, *NBN*, *PALB2*, *RAD51C*, and *RAD51D*. This is because, unlike *BRCA1/2*, there is still substantial debate as to whether the degree of risk of EOC in individuals with GPVs in these genes is sufficiently higher than that in the general population to warrant consideration of RRSO [30]. The unreliability of risk estimates for these genes is primarily attributed to the following factors: the GPV prevalence of candidate genes is generally low; individual ovarian cancer studies typically involve fewer cases than breast cancer studies; and most previous analyses lack a comparable control group, which hinders the interpretation of results [48,49].

The process by which the genome repairs DNA damage from external or internal sources is essential for preventing cell death. One of the most serious DNA alterations can be caused by DSBs, which are lethal to cells if left unchecked [50]. DSBs describe disruptions in both reading frames of the DNA and are often caused by external sources such as ionizing radiation [51]. Two main mechanisms enable cells to repair DSBs: nonhomologous end-joining (NHEJ) and HR [52] (Figure 1). In response to DSBs induced by genotoxic agents in the S/G2 phase, either side of the DNA is lysed from 5′ to 3′ by MRE11. The MRE-RAD50-NBN complex (MRN) is recruited to DSBs and promotes ATM recruitment (Figure 1a). NHEJ causes binding proteins to attach to the open ends of DNA to stabilize and ultimately reconnect the sides of the DNA but does not consider the reading frame, which introduces errors into the DNA [53,54]. ATM phosphorylates and activates many downstream targets that are essential for DNA damage repair via NHEJ and HR (Figure 1b,c). ATM activates other kinases, such as CHEK2, and ultimately phosphorylates multiple proteins that regulate the cell cycle, resulting in cell cycle arrest. This prevents cells from dividing with residual DNA damage, passing DNA damage to daughter cells, and causing chromosomal aberrations. When the amount of DNA damage is large and exceeds the repair capacity of the cell, p53 protein and other proteins are activated to induce cell death or apoptosis. Active ATM creates a platform to recruit BRCA1, which facilitates a shift from NHEJ to HR (Figure 1b).

While this occurs, HR repairs the unaltered reading frame. CtBP-interacting protein, in conjunction with the MRN complex, catalyzes 5′-3′ resection at DSBs to generate single-stranded DNA (ssDNA). From the open ends, a single-strand 3′ opening is created, which allows a series of proteins (e.g., RAD51/BRCA2) to begin searching for a compatible sequence with which to invade and create a D-loop. This process allows both sides to faithfully reconstruct the reading frame [23] (Figure 1c). BRCA1/2 each play multiple, unique roles in HR repair.

For example, BRCA1 is thought to be part of a larger complex molecule that helps to survey DNA for DSB damage [16]. The role of BRCA2 is less clear, but it may play a more direct role in repair by helping the RAD51 complex attach to the repair site. Both BRCA1 and BRCA2 play important roles in a large framework of repair molecules. RAD51 is recruited by the BRCA1–PALB2–BRCA2 effector complex, resulting in their promotion of RPA removal and RAD51 loading [55]. The resulting RAD51-ssDNA filament invades the intact sister chromatid and extends the strand (Figure 1c), which is followed by further restoration and ligation of double strands.

**Figure 2:**
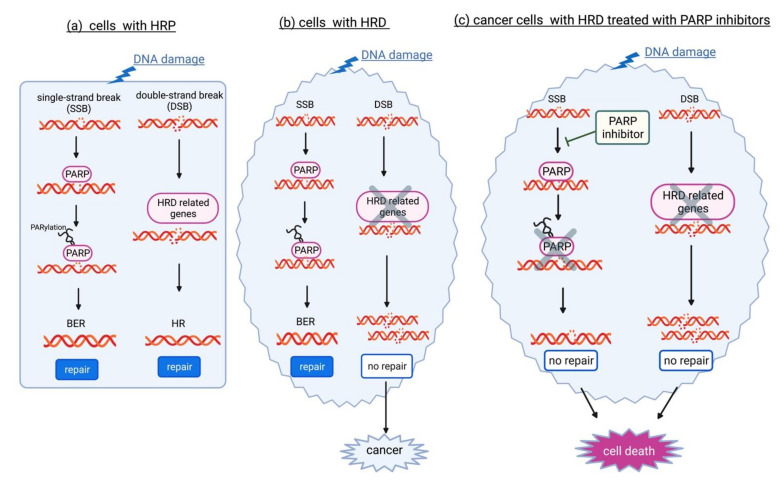
In normal cells which are proficient in homologous recombination (HRP), both base excision repair (BER) and HR are available for the repair of damaged DNA via poly (ADP-ribose) polymerase (PARP) and proteins encoded by HR deficiency (HRD)-related genes, respectively (**a**). In cells with HRD, HR is nonfunctional and leads to carcinogenesis (**b**). When PARP is inhibited by PARP inhibitor, cancer cells with HRD are unable to repair DNA damage by HR and BER, resulting in cell death (synthetic lethality) (**c**).

The multifunctional enzyme Poly ADP ribose polymerase (PARP) plays an important role in DNA damage repair and genome stability. Among the 18 members of the PARP family, PARP-1 is the most important and plays dominant roles in DNA repair pathways. Activated PARP-1 plays an important role in DNA base excision repair (BER) [57]. When PARP-1 fails to function, oxidized bases accumulate. The replication fork stops at the site of the damaged DNA, eventually resulting in DSBs. In normal cells which are proficient at HR (HR proficiency, HPR), both BER and HR are available for the repair of damaged DNA (Figure 2a). Meanwhile, in cells with HR deficiency (HRD), HR is nonfunctional and leads to carcinogenesis (Figure 2b). When PARP-1 is inhibited by PARP inhibitor, cancer cells with HRD are unable to repair DNA damage by HR and BER, leading to cell death (synthetic lethality, Figure 2c). PARP-1 is also involved in the regulation of nucleotide excision repair (NER), classical NHEJ (cNHEJ), alternative NHEJ (aNHEJ), microhomology-mediated end-joining (MMEJ), HR, MMR, and maintenance of replication fork stability [58]. For example, PARP-1 recruits DSB repair enzymes MRE11 and NBS1 for modulating DSB repair [59].

PARP inhibitors are the first clinically approved anti-cancer agents which specifically targeted the DNA damage response in *BRCA1/2*-mutated cancers [60]. In advanced-stage EOC patients with the *BRCA1/2* GPVs, maintenance therapy with a PARP inhibitor (olaparib) resulted in a 70% lower risk of disease progression or death (SOLO1 clinical trial) [55]. The phase III OlympiAD trial showed that olaparib had better efficacy than standard chemotherapy for HER2-negative breast cancer patients with the *BRCA1/2* GPVs [61]. Their indication has been gradually extended to prostate and pancreatic cancer patients with *BRCA1/2* GPVs [56]. More recently, the effect of PARP inhibitors was also validated in HR-deficient cancers without BRCA*1/2* GPVs, suggesting widespread use of PARP inhibitors for cancers caused by GPVs in other HR pathway genes [62,63].

## 3. *ATM* (Ataxia–Telangiectasia Mutated) Gene

### 3.1. Molecular Function in the Response to DSBs

The *ATM* gene encodes a protein kinase with pleiotropic functions belonging to the superfamily of phosphatidylinositol 3-kinase-related protein kinases at the peak of a cascade responding to DSBs [64]. In DSB repair, the HR repair pathway is largely restricted to the S and G2 phases of the cell cycle, when an intact sister chromatid is available as a template, whereas NHEJ can be active in any cell cycle [65] (Figure 1b). ATM, which is recruited and activated by the MRN protein complex that recognizes the free DNA ends of DSBs, phosphorylates many important proteins, e.g., BRCA1, p53, AKT, and CHEK2 proteins, thereby mediating the DNA damage response, promoting cell cycle arrest, or inducing apoptosis. In addition to playing a key role in HR, ATM also orchestrates DSB repair by preventing the toxic error-prone NHEJ pathway [66,67]. *ATM* GPV heterozygous carriers have an increased risk for several types of cancers, including breast, ovarian, and pancreatic cancers [46,68]. However, the carriers of biallelic *ATM* GPVs are affected by ataxia–telangiectasia (AT, OMIM #208900), which is a rare autosomal recessive syndrome characterized by progressive cerebellar ataxia, cutaneous telangiectasias, increased risk of developing hematologic and solid tumors, and immunodeficiency [66,68,69].

### 3.2. Prevalence and Risk of Developing EOC

A recent meta-analysis [45] reported the prevalence of *ATM* GPVs in patients with EOC to be 0.6767% (26/3842 cases) and showed a significant association between *ATM* GPVs and EOC (odds ratio (OR) = 1.977, 95% confidence interval (CI) = 1.330–2.939) (Table 1). Another population-based cohort study reported that the prevalence of *ATM* GPVs was 0.57–0.64% [18,69]. The absolute lifetime risk of EOC estimated by the NCCN clinical practice guidelines in oncology is <3% [26,46].

### 3.3. Medical Management for the Prevention of EOC

For heterozygote *ATM* GPV carriers, there is insufficient evidence available to recommend RRSO, although a large EOC study reported strong evidence for an approximately two-fold increased risk of developing EOC compared with noncarriers [46]. Therefore, RRSO should be considered according to the family history of the patient (Table 1) [46]. The detection of heterozygous *ATM* GPVs should not lead to a recommendation to avoid radiation therapy at this time [46]. Furthermore, the NCCN clinical practice guidelines in oncology recommend counseling for *ATM* GPV carriers because of the risk of autosomal recessive inheritance in their offspring [46].

## 4. *BRIP1* (BRCA1 Interacting Helicase 1) Gene

### 4.1. Molecular Function in the Response to DSBs

The protein encoded by *BRIP1* is a member of the RecQ DEAH helicase family and part of the Fanconi anemia group. The BRIP1 protein interacts with the BRCT repeats at the carboxyl-terminus of BRCA1 (Figure 1b). The bound complex is important for normal DSB repair by HR. BRIP1 is also physiologically essential for maintaining genomic integrity, removing proteins bound to DNA, stabilizing replication forks, and unwinding substitutive DNA structures along with RPA [70].

### 4.2. Prevalence and Risk of Developing EOC

GPVs in *BRIP1* are the second most common pathogenic variant found in patients with EOC after those in *BRCA1/2*, with a frequency of approximately 1% of EOC cases (Table 1) [17,45]. In a recent meta-analysis [46], *BRIP1* GPVs were significantly associated with EOC (OR = 4.878, 95% CI = 3.729–6.380). Another population-based cohort study reported that the prevalence of *BRIP1* GPVs was 0.92–1.36% [18,71]. A larger meta-analysis using approximately 29,400 EOC cases from 63 studies and approximately 116,000 controls from the gnomAD database reported that the prevalence of *BRIP1* GPVs in patients with EOC was 0.8891% (200/22,494 cases) and that BRIP1 was significantly associated with EOC (OR = 4.94, 95%CI = 4.07–6.00) [49]. The NCCN clinical practice guidelines in oncology estimate that the absolute lifetime risk of EOC for individuals with *BRIP1* GPVs is >10% [46].

### 4.3. Medical Management for the Prevention of EOC

For *BRIP1* GPV carriers, the NCCN clinical practice guidelines in oncology recommend that RRSO should be considered from age 45 to 50 years or earlier based on a specific family history of early-onset EOC [45,46,72] (Table 1). Although the lifetime risk of EOC in *BRIP1* GPV carriers seems to be sufficient to justify considering RRSO, there is currently no evidence to make a firm recommendation on the optimal age for this procedure. Reportedly, the median age at diagnosis for *BRIP1* GPV carriers with EOC is 65 years old [72]. Moreover, the age at which to begin consultation for surgery may change as more evidence is collected. Furthermore, because *BRIP1* was originally identified in research on Fanconi anemia (FANCJ; OMIM #609054) [73], the NCCN clinical practice guidelines in oncology recommend counseling *BRIP1* GPV carriers about the risk of autosomal recessive conditions in their offspring [46].

## 5. *NBN* (Nibrin) Gene

### 5.1. Molecular Function in the Response to DSBs

*NBN* encodes the protein NBN or nibrin, one of the components of the MRN protein complex, which is essential for DSB repair, DNA recombination, maintenance of telomere integrity, cell cycle checkpoint regulation, and meiosis (Figure 1a) [74]. The MRN protein complex is composed of two heterodimers of RAD50 and MRE11, as well as a single NBN, and possesses single-strand endonuclease activity and double-strand-specific 3′-5′ exonuclease activity provided by MRE11. In DSB repair, RAD50 is required to bind DNA ends and hold them in close proximity [75]. NBN modulates DNA damage signal sensing by recruiting ATM, ATR, and DNA-dependent protein kinase catalytic subunits to the sites of DNA damage and activating their functions [76]. NBN can also recruit MRE11 and RAD50 to the proximity of DSBs via its interaction with the histone H2AX [77]. NBN also functions in telomere length maintenance by generating the 3′ overhang which serves as a primer for telomerase-dependent telomere elongation [78].

GPVs at the homozygous or compound heterozygous status within *NBN* are responsible for Nijmegen breakage syndrome (NBS; OMIM #251260), a rare autosomal recessive disorder characterized by microcephaly, growth retardation, humoral and cellular immunodeficiency, radiosensitivity, and cancer predisposition. By the age of 20 years, more than 40% of patients with NBS develop a malignant disease, primarily of lymphoid origin [79].

### 5.2. Prevalence and Risk of Developing EOC

As NBN has an essential function in the DNA repair pathway, several case–control studies have investigated its status as an EOC susceptibility gene. However, most studies have provided insufficient evidence of a significant association with the risk of developing EOC [42,46]. In a recent meta-analysis [45], the reported prevalence of *NBN* GPVs in patients with EOC was 0.2837% (20/7050 EOC cases), and *NBN* GPVs were significantly associated with EOC (OR = 2.166, 95% CI = 1.346–3.488) (Table 1). Another population-based cohort study including 6,001 patients with EOC reported a prevalence of *NBN* GPVs of 0.35% [71]. According to the NCCN clinical practice guidelines in oncology, the absolute lifetime risk of EOC in *NBN* GPV carriers is relatively low (<3%); however, the evidence strength is limited, and insufficient data are available [46].

### 5.3. Medical Management for the Prevention of EOC

According to the NCCN clinical practice guidelines in oncology, there is currently insufficient evidence to recommend RRSO in *NBN* GPV carriers at this time [46]. Medical management for EOC risk should be considered based on family history [46]. Because the *NBN* gene is associated with the development of NBS, the NCCN clinical practice guidelines in oncology recommend counseling *NBN* GPV carriers about the risk of autosomal recessive conditions in their offspring [46].

## 6. *PALB2* (Partner and Localizer of *BRCA2*) Gene

### 6.1. Molecular Function in the Response to DSBs

*PALB2* was originally identified as the gene encoding protein immunoprecipitated with the BRCA2 protein. PALB2 colocalizes with BRCA2 in nuclear foci; promotes the stable association of BRCA2 with nuclear structures, allowing BRCA2 to escape the effects of proteasome-mediated degradation; and enables the HR repair of DSBs and the maintenance of G2/M checkpoint functions (Figure 1b) [80,81].

### 6.2. Prevalence and Risk of Developing EOC

Although the previous NCCN clinical practice guidelines in oncology described “ovarian cancer risk and management” for *PALB2* GPVs as insufficient evidence (ver1.2022), the latest version (ver2.2022) has been updated to state that the evidence is strong [46]. A recent meta-analysis reported that the prevalence of *PALB2* GPVs in patients with EOC was 0.4226% (30/7099 EOC cases), and that *PALB2* GPVs were significantly associated with EOC (OR = 2.134, 95% CI = 1.420–3.207) (Table 1) [45]. However, the relationship between *PALB2* GPVs and EOC susceptibility is debated and exhibits low statistical significance. Another population-based cohort study reported that the prevalence of *PALB2* GPVs was 0.40–0.62% [18,71].

A recent international study of 524 families with *PALB2* GPVs estimated the relative and cumulative risks using complex segregation analysis to model the cancer inheritance patterns in families while adjusting for the mode of ascertainment of each family [82]. This study demonstrated that the estimated risk of female *PALB2* GPV carriers developing EOC by the age of 80 was 5%. Based on this result, the NCCN clinical practice guidelines in oncology estimate an absolute lifetime risk of EOC in heterozygote *PALB2* GPV carriers of 3–5%, with strong evidence [46].

### 6.3. Medical Management for the Prevention of EOC

Although ACMG guidance showed that *PALB2* GPV carriers had a small to moderate risk for EOC [83], the clinical benefit of RRSO was not sufficiently proven to reduce morbidity and mortality. For all *PALB2* GPV carriers, there is insufficient evidence available to recommend RRSO. Therefore, RRSO should be considered based on family history for EOC (Table 1) [46]. As *PALB2* is a Fanconi anemia gene (FANCN; OMIM #610832), the NCCN clinical practice guidelines in oncology recommend counseling *PALB2* GPV carriers about the risk of autosomal recessive conditions in their offspring [46].

## 7. RAD51C/RAD51D Gene

### 7.1. Molecular Function in the Response to DSBs

*RAD51C* and *RAD51D* encode the RAD51 paralog proteins, RAD51C and RAD51D, which are structurally similar to the RAD51 recombinase. The Rad51 paralogs associate with one another in two distinct complexes: RAD51B-RAD51C-RAD51D-XRCC2 (BCDX2) and RAD51C-XRCC3 (CX3) [56]. The RAD51 paralogs participate in the assembly and stabilization of the ssDNA/RAD51 filament and the HR intermediates. They are also involved in the process downstream of the homology search.

### 7.2. Prevalence and Risk of Developing EOC

A recent meta-analysis [45] reported that the prevalence of *RAD51C* and *RAD51D* GPVs in patients with EOC was 0.5539% (21/3791 EOC cases) and 0.5832 (19/3258 EOC cases), respectively, and that *RAD51C* and *RAD51D* were significantly associated with EOC (OR = 4.241, 95% CI = 2.562–7.022, and OR = 7.276, 95% CI = 4.028–13.140, respectively) (Table 1). Another population-based cohort study reported that the prevalence of *RAD51C* and *RAD51D* GPVs was 0.57% and 0.57%, respectively [18]. In a larger meta-analysis using 29,400 EOC cases and 116,000 controls from the noncancer gnomAD database, the prevalence of *RAD51C* and *RAD51D* GPVs with EOC was 0.6260% (149/23,802 cases) and 0.4125% (94/22,787 cases), respectively, and *RAD51C* and *RAD51D* were significantly associated with EOC (OR = 5.59, 95%CI = 4.42–7.07 and OR = 6.94, 95%CI = 5.10–9.44, respectively) [48].

A recent study including 6,178 and 6,690 families with known *RAD51C* and *RAD51D* GPVs, respectively, estimated the relative and cumulative risks using complex segregation analysis to model the cancer inheritance patterns in families while adjusting for the mode of ascertainment of each family [84]. According to the results of this relatively large case–control study, the cumulative risk of developing EOC by the age of 80 years was 11% and 13% for *RAD51C* and *RAD51D* GPV carriers, respectively. Thus, the NCCN clinical practice guidelines in oncology estimate the absolute lifetime risk of EOC in heterozygote *RAD51C/RAD51D* GPV carriers as >10% [46].

### 7.3. Medical Management for the Prevention of EOC

For *RAD51C/RAD51D* GPV carriers, the NCCN clinical practice guidelines in oncology recommend considering RRSO from age 45 to 50 years or earlier based on a specific family history of early-onset EOC [46,72]. Although the lifetime risk of EOC in *RAD51C/RAD51D* GPV carriers seems to be sufficient to justify considering RRSO, there is insufficient evidence to make a firm recommendation regarding the optimal age for this procedure. Reportedly, the median age at diagnosis for *RAD51C/RAD51D* GPV carriers with EOC is 62 and 57 years old [72]. Therefore, the age at which to begin consultation for surgery may change as more evidence is accumulated. As *RAD51C* is a Fanconi anemia gene (FANCO; OMIM # 613390), the NCCN clinical practice guidelines in oncology recommend counseling *RAD51C* GPV carriers about the risk of autosomal recessive conditions in their offspring [46].

*RAD51C*- [85] and *RAD51D*-deficient [86] cells, or those expressing pathogenic variants in these genes [86,87], have been shown to render sensitivity to PARP inhibitors, such as olaparib, which is the first PARP inhibitor to be approved for EOC treatment [62,63,88,89,90]. However, it remains unclear whether identifying *RAD51C/RAD51D* GPVs in patients with EOC is useful for identifying patients that might benefit from treatment with protocols using PARP inhibitors [50].

## 8. Conclusions

Compared to other cancers, EOC includes a relatively high percentage of hereditary tumors. Approximately 50% of patients with hereditary EOC harbor GPVs in the *BRCA1/2* genes contributing to the HR repair pathway. Moreover, other genes participating in the HR repair pathway, such as *ATM*, *BRIP1, NBN*, *PALB2*, and *RAD51C/D*, are also known as predisposition genes related to hereditary EOC with moderate penetrance. This review has outlined the current knowledge of these moderate-risk genes for EOC involved in the HR repair pathway. In addition to the molecular functions of these EOC-associated genes, we discussed the recommended clinical strategies for preventing EOC in individuals carrying GPVs in these genes. This review can improve our ability to estimate the genetic risk of developing EOC and select appropriate preventive and treatment strategies for hereditary EOC.

However, there are still some issues limiting the effective medical management for EOC based on individual genetic analysis. First, the penetrance of hereditary tumors is not 100%, and the significance of detecting moderate-risk genes for medical management remains unclear [91]. Second, further data registration is important because the GPV frequency prevalence of each cancer predisposition gene varies among populations. Third, understanding the relationship between genotype and phenotype may be extremely useful in a clinical setting. However, if the results are not properly interpreted and explained, there is a risk of inappropriate treatment. Fourth, along with the education of clinicians, it is crucial to ensure collaboration among clinicians, researchers, and companies that provide genetic testing for providing medical care based on genetic information.

## Figures and Tables

**Table 1:** Frequency of germline pathogenic variants in patients with epithelial ovarian cancers (EOC), relative and absolute risks for EOC, and risk reduction for EOC in each predisposition gene.

Gene	Suszynska et al. [45]			NCCN Guidelines [46,47]		
	Frequency of GPV in EOC Patients (%)	Relative Risk for EOC		Absolute Risk for EOC	Evidence for Association	Management for Risk Reduction
		OR (95% CI)	*p*-Value			
*BRCA1*	8.607	35.26 (29.56–42.05)	<0.0001	39–58%	very strong	RRSO recommended for patients aged 35–40 yrs
*BRCA2*	4.520	11.91 (9.87–14.39)	<0.0001	13–29%	very strong	RRSO recommended for patients aged 40–45 yrs
*BRIP1*	1.057	4.88 (3.73–6.38)	<0.0001	>10%	strong	RRSO considered for patients aged 45–50 yrs
*CHEK2*	0.703	0.43 (0.29–0.63)	<0.0001	not established	not established	not established
*ATM*	0.677	1.98 (1.33–2.94)	0.001	<3%	insufficient	manage based on family history
*RAD51C*	0.554	4.24 (2.56–7.02)	<0.0001	>10%	strong	RRSO considered for patients aged 45–50 yrs
*RAD51D*	0.583	7.28 (4.03–13.14)	<0.0001	>10%	strong	RRSO considered for patients aged 45–50 yrs
*MSH6*	0.444	4.08 (2.43–6.85)	<0.0001	<13%	insufficient, mixed	-
*PALB2*	0.423	2.13 (1.42–3.21)	0.0003	3–5%	insufficient	manage based on family history
*TP53*	0.294	5.05 (2.41–10.58)	<0.0001	not established	not established	not established
*NBN*	0.284	2.17 (1.35–3.49)	0.0020	insufficient data	limited	manage based on family history
*MSH2*	0.238	3.98 (1.18–8.69)	0.0007	>10%	strong	RRSO should be individualized after childbearing
*PMS2*	0.183	0.71 (0.29–1.72)	0.5633	<3%	limited	-
*MLH1*	0.104	1.44 (0.53–3.91)	0.6815	>10%	strong	RRSO should be individualized after childbearing
*BARD1*	0.142	1.41 (0.69–2.89)	0.4706	not established	not established	not established
*PTEN*	0.063	5.47 (1.26–23.82)	0.0799	not established	not established	not established

CI, confidence interval; EOC, epithelial ovarian cancer; GPV, germline pathogenic variant; OR, odds ratio; RRSO, risk-reducing salpingo-oophorectomy; yrs, years. Genes in boldface indicate those described in this review article.

## Data Availability

Not applicable.
